# Does gated beam delivery impact delivery accuracy on an Elekta linac?

**DOI:** 10.1002/acm2.12020

**Published:** 2017-01-03

**Authors:** Mohammed Jermoumi, Roger Xie, Daliang Cao, David J. Housley, David M. Shepard

**Affiliations:** ^1^ Department of Radiation Oncology Swedish Cancer Institute Seattle WA USA; ^2^ Department of Radiation Oncology Ironwood Cancer and Research Center Scottsdale AZ USA

**Keywords:** beam delivery accuracy, linac gating performance, respiratory gating, VMAT

## Abstract

In this study, we evaluated the performance of an Elekta linac in the delivery of gated radiotherapy. Delivery accuracy was examined with an emphasis on the impact of using short gating windows (low monitor unit beam‐on segments) or long beam hold times. The performance was assessed using a 20cm by 20cm open field with the radiation delivered using a range of beam‐on and beam‐off time periods. Gated delivery measurements were also performed for two SBRT plans delivered using volumetric modulated arc therapy (VMAT). Tests included both free‐breathing based gating (covering a variety of gating windows) and simulated breath‐hold based gating. An IBA MatriXX 2D ion chamber array was used for data collection, and the gating accuracy at low MU was evaluated using gamma passing rates. For the 20 cm by 20 cm open field, the measurements generally showed close agreement between the gated and non‐gated beam deliveries. Discrepancies, however, began to appear with a 5‐to‐1 ratio of the beam‐off to beam‐on times. The discrepancies observed for these tight gating windows can be attributed to the small number of monitor units delivered during each beam‐on segment. Dose distribution analysis from the delivery of the two SBRT plans showed gamma passing rates (± 1%, 2%/1 mm) in the range of 95% to 100% for gating windows of 25%, 38%, 50%, 63%, 75%, and 83%. Using a simulated sinusoidal breathing signal with a 4 second period, the gamma passing rate of free‐breathing gating and breath‐hold gating deliveries were measured in the range of 95.7% to 100%. In conclusion, the results demonstrate that Elekta linacs can accurately deliver respiratory gated treatments for both free‐breathing and breath‐hold patients. Some caution should be exercised with the use of very tight gating windows.

## 1 Introduction

Normal diaphragmatic excursion during uncontrolled breathing can result in significant respiratory‐induced motion for tumors of the lung and liver. In radiation therapy, the impact of respiratory motion is typically accounted for by creating a target volume which fully encompasses the tumor movement. This approach, however, can result in large volumes of non‐target tissue being irradiated. This can increase the toxicity of treatment and limit the dose that can be delivered to the tumor. Researchers have developed alternative techniques that account for respiratory motion to reduce the target volume. These techniques include tumor tracking and gated beam delivery.^1^, ^2^, ^3^, ^4^, ^5^, ^6^, ^7^, ^8^

Gated beam delivery has the advantage of being less technically complex as compared to multileaf collimator (MLC) based tracking. The downside of a gated approach, however, is decreased treatment efficiency that results in longer treatment times.^9^ In gated beam delivery, the linear accelerator beam is typically triggered on and off at either full‐inspiration or end‐expiration. The user determines a gating window, and radiation is only delivered during a specified phase of the breathing cycle.^10^ One common approach to gated beam delivery is the use of a deep‐inspiration breath hold (DIBH) technique for left‐sided breast cancers with a goal of minimizing the dose to the heart and lung.^9^ Gated delivery is also used in the treatment of solid lung cancers.^11^, ^12^

When commissioning a system for gated radiotherapy, it is important to characterize the startup characteristics of the accelerator.^13^ This is true because gated radiotherapy introduces delivery situations not typically encountered in external beam radiotherapy. With free‐breathing gating, the radiation is delivered using a large number of segments. The use of a tight gating window combined with beam‐on delays can result in a low number of monitor units (MUs) per deliverable segment. Previous studies have demonstrated the need to characterize beam stability for short irradiation times.^14^, ^15^, ^16^ Additionally, for breath‐hold‐based gating, the beam is held for an extended period between each delivery segment. The impact of these prolonged beam‐holds on the accuracy of the delivered radiation needs to be addressed.

Gated delivery techniques have been investigated for Varian linacs.^17^, ^18^ More recently, the gating characteristics (e.g., beam profile and beam delivery efficiency) have been evaluated for Elekta Precise and Synergy linacs.^10^, ^19^ In this study, we have focused on the beam startup characteristics for gated delivery of an Elekta linac and the overall accuracy of the delivery for a variety of gating scenarios.

Using an Elekta Synergy linac (Elekta AB, Stockholm, Sweden) in our clinic, gated beam delivery was performed using an Elekta Response gating interface. The Response gating kit received 510(k) clearance in 2013 and for the first time provided a tool to gate Elekta linacs in an automated manner. Previously, tools like the Active Breathing Coordinator (ABC) required a manual gating process where the ABC unit operated independent of the linac. The therapist would manually gate the beam on and off for this breath‐hold based gating technique.

For this study, we wanted to test whether the delivery accuracy would be compromised if the delivery utilized a tight gating window that resulted in the delivery of a low number of monitor units for each breathing cycle. We performed tests to validate the gated beam delivery accuracy while assessing a variety of gating windows. Comparisons were made between the gated delivery and non‐gated delivery (baseline). Deliveries were also performed without the use of the Elekta Response Gating kit. For these deliveries, each segment was delivered as a separate beam meaning the beam was not coming out of an active hold when it turned on. The gated technique was evaluated using two clinical plans that were delivered under free‐breathing (FB) and breath‐hold (BH) modes using a simulated breathing pattern.

## 2 Methods

### 2.A Beam delivery characteristics

**Figure 1:**
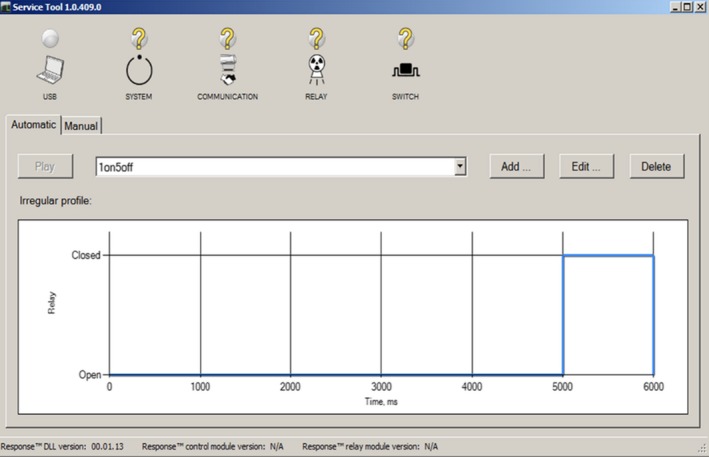
User interface of the Response kit gating software (Elekta AB, Stockholm, Sweden) used to simulate a variety of scenarios for beam‐on/off to perform automatic gating beam delivery using a square wave. The figure shows the signal pattern for a beam‐on of 1 second and beam‐off of 5 seconds.

The gated beam delivery was triggered using the Elekta Response gating interface (Elekta AB, Stockholm, Sweden). The Elekta Response gating interface consists of a gating switch box that enables or disables the gated beam delivery. Gating signals were created using gating control software (Elekta AB, Stockholm, Sweden) that uses a digital signal (0 for beam‐off and 1 for beam‐on) to simulate free‐breathing and breath‐hold signals (Fig. 1). Gated beam deliveries were performed using a number of beam‐on and beam‐off combinations.

In Elekta's linear accelerator delivery control software, the user can set the maximum gun‐hold time. If the delay time between beam segments in a delivery exceeds the specified maximum gun‐hold time, the linac switches from an active mode to a standby mode. For this work, the maximum gun‐hold time was set to the highest allowable value of 6.5 seconds.^10^ The advantage of setting a long gun‐hold time is that the beam‐on delays are significantly less when the beam is turned on out of an active beam‐hold state. This results in a more efficient delivery. One of the goals of this work was to determine if there is any loss in dosimetric accuracy by setting the gun‐hold value to the maximum allowed value. In other, words does setting up the system in a manner that maximizes delivery efficiency have negative consequences in terms of delivery accuracy?

A 20 × 20 cm^2^ open field (with 20 MU or 200 MU deliveries) was used to test the gating accuracy. First, the field was delivered in a normal mode (N mode). Next, the same field was delivered using the gated beam delivery mode (G mode). The gating windows were defined with beam‐on times of 1, 3, and 5 seconds and beam‐off times of 1, 3, and 5 seconds (Tables 1 and 3).

A second technique for delivering the open field was tested using a series of separately delivered segments each assigned a small number of MUs (M mode). This approach mimics the delivery mechanism used when a small gun‐hold time is set in the delivery control software. It also mimics the manual gating approach that was employed with the active breathing coordinator (ABC) device prior to the availability of the response gating kit. For example, we can deliver a 20 × 20 cm^2^ field with 4 MU five times to achieve the same effect as delivering a single 20 MU field. As compared with the gated delivery where beam was held between each gating window, such a delivery requires the beam to switch on and off for each radiation delivery. For gating tests using actual patient treatment plans, two SBRT VMAT cases were used.

### 2.B Phantom measurements

In the gated and static beam deliveries, the dose measurements were performed with an IBA MatriXX Evolution 2D ion chamber array inserted into a MultiCube phantom (IBA Dosimetry, Schwarzenbruck, Germany). The detector array has an active measurement area of 24 × 24 cm^2^ and contains 1024 micro ion‐chambers. The gated beam delivery using a variety of gating windows was carried out at 6 MV, 10 MV, and 18 MV (Table 1). For beam delivery using M mode, the measurements were performed at 6 MV. A dose delivery was performed using an Elekta Synergy with nominal dose rates of 450, 400, and 600 MU/min for 6 MV, 10 MV, and 18 MV, respectively.

**Table 1:** Reproducibility of measurements using the IBA MatriXX detector array for gating and nongating beam delivery. All measurements were performed at 6 MV with 20 MU beam delivery

	Beam‐on/off time (s)	1%, 1 mm			2%, 1 mm		
		Mean	Std	CV (%)	Mean	Std	CV (%)
Gating mode	(1:1)	98.53	1.33	1.36	100.00	0.00	0.00
	(1:3)	98.07	1.67	1.72	100.00	0.01	0.00
	(1:5)	97.34	0.59	0.78	99.68	0.22	0.00
	(3:1)	100.00	0.00	0.00	100.00	0.00	0.00
	(3:3)	100.00	0.00	0.00	100.00	0.00	0.00
	(3:5)	100.00	0.00	0.00	100.00	0.00	0.00
	(5:1)	100.00	0.00	0.00	100.00	0.00	0.00
	(5:3)	100.00	0.00	0.00	100.00	0.00	0.00
	(5:5)	100.00	0.00	0.00	100.00	0.00	0.00
Non gating mode	Open field	100.00	0.00	0.00	100.00	0.00	0.00

### 2.C SBRT patient treatment plan and simulated natural breathing motion

The gated beam delivery accuracy was evaluated using two lung SBRT VMAT plans. Both plans used two 360‐degree arcs along with a 6 MV beam to deliver 1200 cGy per fraction. The VMAT plans were generated with the Pinnacle^3^ treatment planning system (Philips Medical, Madison, WI, USA). Using the Response kit, respiratory motion was simulated with a breathing period of 4 seconds in free‐breathing (FB) mode with a number of beam‐on to beam‐off combinations (1:3), (1.5:2.5), (2:2), and (3:1) (Table 4) and beam‐on/off times of 6 and 12 seconds to simulate a breath‐hold (BH) scenario (Table 4).^16^

### 2.D Data analysis

In this work, the results from G mode and M mode were compared to the result of N mode to assess the gating delivery accuracy. The OmniPro‐I'mRT 1.5a (IBA Dosimetry, Schwarzenbruck, Germany) software was used to analyze the collected data based on gamma index evaluation and using the movie mode with a frame rate of 0.1 seconds. A dose grid was converted to spacing of 7.6 mm using linear interpolation. The passing rates using gamma index criteria of 1% and 2% with ± 1 mm distance‐to‐agreement (DTA) were determined for all measurements.^20^

## 3 Results

### 3.A Measurement reproducibility

The reproducibility of the measurements performed with the MatriXX array detector was determined for gating and nongating mode (Table 1). The mean, standard deviation, and coefficient of variation were determined for three trials. Using a gamma score (1%/1 mm), the percentage coefficient of variation (CV) was less than 2% for G mode, and no statistically significant variation was observed for the open field (N mode). Using a gamma score of 2%/1mm, the measurement variation approached zero.

### 3.B Dose distribution comparison of gated and non‐gated beam delivery at 20 and 200 MU

For 6 MV, excellent agreement was observed between the G mode and the N mode. The gamma passing rates were greater than 99% for all of the gating windows and energies using 1 mm and 2% criteria. These findings were comparable to those obtained using a step‐and‐shoot delivery technique.^13^ However, with stricter agreement criteria of 1%/1mm, the gamma score decreased slightly to 97.66% (Table 2) for 6 MV and ~92% for 10 MV and 18 MV.

**Table 2:** Gamma score (1%/1 mm; 2%/1 mm) for gated and non‐gated beam delivery for 20 MUs and 200 MUs using open field of 20 cm by 20 cm. The beam‐on/off time is represented by (m:n) where the m is beam‐on time and n is beam‐off time for a gated delivery

Duty cycle (%)	50	25	17	75	50	38	83	63	50
Time (s)	(1:1)	(1:3)	(1:5)	(3:1)	(3:3)	(3:5)	(5:1)	(5:3)	(5:5)
6 MV (20 MU)									
1%	99.54	98.73	98.49	99.69	99.93	99.59	100	100	99.99
2%	100	100	100	100	100	100	100	100	100
6 MV (200 MU)									
1%	99.95	98.82	97.66	100	100	100	100	100	99.99
2%	100	100	100	100	100	100	100	100	100
10 MV (20 MU)									
1%	98.28	98.57	92.42	100	100	100	100	100	100
2%	100	100	99.9	100	100	100	100	100	100
18 MV (20 MU)									
1%	95	95	91.8	100	100	100	100	100	100
2%	100	100	100	100	100	100	100	100	100

### 3.C Dose distribution comparison of multistatic beam delivery with small MU (M mode) and single static delivery of large MU (N mode) at 20 and 200 MU

A dose distribution comparison between M mode and N mode beam deliveries showed a high level of agreement when each beam was delivered with more than 10 MU. The M mode delivery showed significant degradation of the beam quality with a much lower gamma passing rate for radiation delivery with a small number of monitor units per segment (4 MU and 2 MU) as seen in Table 3.

**Table 3:** Gamma score (1%/1 mm; 2%/1 mm) for dose distribution for static beam delivery of segmented and non‐segmented beam for 20 MU and 200 MU measured at 6 MV with open field of 20 cm by 20 cm

20MU	10MU × 2	4MU × 5	2MU × 10
1%	99.88	55.59	24.87
2%	100	88.42	52.41

**200 MU**	**100 MUx2**	**40 MUx5**	**20 MUx10**
1%	100	100	93.29
2%	100	100	99.23

### 3.D Gated beam delivery in FB and BH mode using VMAT plan of SBRT

When 2%/1 mm passing criteria were used, all gated SBRT VMAT deliveries had gamma passing rates greater than 95% for all gating scenarios. However, with a ± 1% tolerance, the lowest gamma scores were 69.42 and 66.74 for patient A and B, respectively, when using a gating window of 17% (1s:5s) (Table 4) which agrees with the finding obtained using an open field (Table 2). For other cases, the results fell within a range of 95% to 99%. Additionally, with the FB and BH modes, the gamma passing rates were between 95% and 100% for both patients (Table 4).

**Table 4:** Gamma score (1%/1mm; 2%/1mm) for gated beam delivery for patient A and B using selected beam‐on/off time delivery. Gated beam delivery for FB mode with the beam‐on/off time of (1:3), (1:5), (2:2), and (3:1) and for BH mode with the beam‐on/off time of (12:6)

Duty cycle (%)	50	25	37.5	20	17	75	50	50	38	83	63	50	66.6
Time (s)	(1:1)	(1:3)	(1.5:2.5)	(1:4)	(1:5)	(3:1)	(2:2)	(3:3)	(3:5)	(5:1)	(5:3)	(5:5)	(12:6)
1%	99.82	95.72	97.43	96.57	69.42	99.91	99.56	96.62	96.43	99.93	98.27	97.69	100
2%	100	99.69	99.92	100	99.47	100	100	99.96	99.99	100	100	100	100
1%	97.61	98.83	99.16	98.91	66.74	99.42	99.35	99.06	99.98	99.94	99.23	99.3	100
2%	100	100	100	100	96.9	100	100	100	100	100	100	100	100

## 4 Discussion

We investigated the gated beam delivery accuracy when a small number of monitor units are delivered in each gating window. The findings regarding measurement reproducibility were similar to those reported by Elizabeth et al.^21^ Close agreement in dose distribution comparisons were found for both gated and non‐gated beam deliveries using both an open field and VMAT delivery techniques. When the gating window was reduced to 17% (1 second on, 5 seconds off), a reduced dosimetric accuracy was observed (Table 1). Additionally, the gamma passing rate was also lower for the higher beam energy of 18 MV. When the gated beam delivery was performed using VMAT, the gamma score results became significantly lower for the tightest gating window (17%). In fact, the impact of the beam‐on delay became more pronounced for VMAT delivery as compared with open fields.^10^ The VMAT delivery was characterized by considerable cumulative beam on delays caused by complex nature of the delivery with the gantry and leaf speed motion combined with the gated beam delivery.^10^, ^22^ Therefore, care must be taken when using a tight gating window of (e.g., 17% or less) to ensure the accuracy of the delivered dose.

Using multistatic beam delivery (M mode) where the MUs delivered were small (four or less), the gamma passing rate decreased dramatically. Switching the beam‐on/off with small MUs, deteriorates the performance of the linac. In fact, the radiation beam could not reach a stable state for the first few MUs. This may be due to the effect of temperature change on the magnetron and the gun current.^14^ As result, the beam delivery accuracy with small monitor unit segments could be negatively impacted.

**Figure 2:**
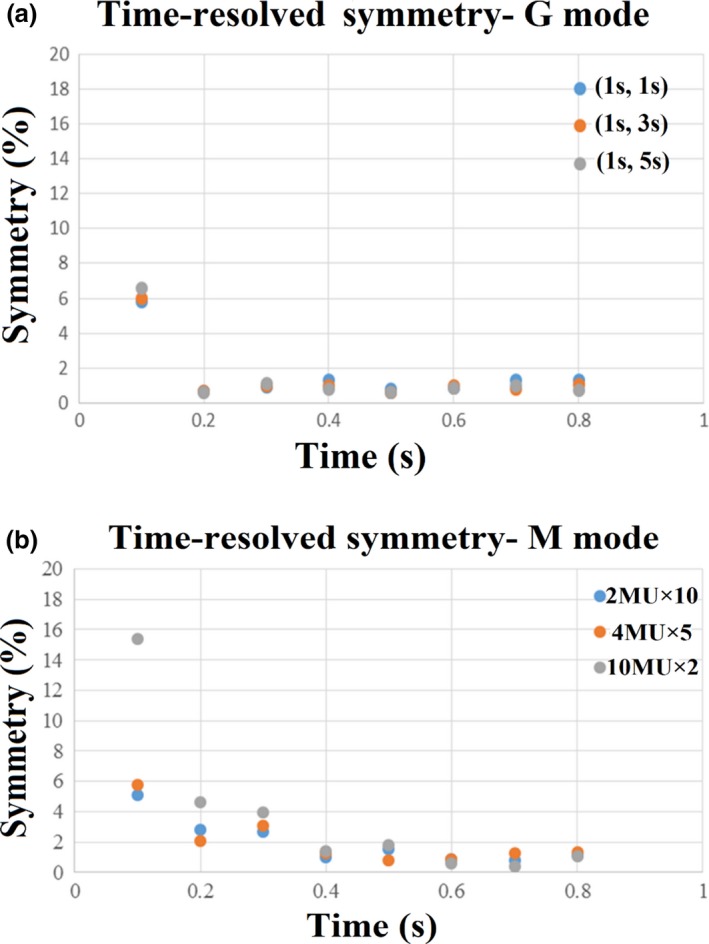
An example of time‐resolved symmetry for gated beam delivery of 20 MU. (a) using beam‐on/off time of (1 s:1 s), (1 s:3 s), and (1 s:5 s) and (b) static beam delivery of 20 MU using segment of 2MU × 10, 4MU × 5, and 10MU × 2.

Figure 2 shows the time‐resolved profile symmetry. It can be seen that the G mode delivery reaches a stable beam symmetry more rapidly as compared with the M mode beam delivery. To reach profile symmetry stability, the M mode needed 0.4 seconds compared to 0.2 seconds with G mode. Within the first second of radiation delivery, over 87% of dose profile symmetry points agreed within 2% for gated and 63% for static beam delivery. Thus, the gated beam‐on hold is able to reach a stable state more quickly than starting a beam from the beam‐off state. These results demonstrate improved dosimetric accuracy using gated beam delivery relative to the multistatic beam technique when a small number of MUs are delivered.

With an Elekta linac, the electron gun voltage will jump from standby state to active state when a static beam is delivered. After the prescribed MUs are delivered, the electron gun voltage returns back to the standby state. This is different in gated beam delivery where the electron gun will remain in an active state during the beam hold when the gating signal is outside the gating window. The findings of this work could serve as a starting point to develop a quality assurance protocol for gated beam delivery using an Elekta linac. In the meantime, another validation study could be performed using film dosimetry or linac log files for dose verification during gated and interrupted beam delivery.^23^

## 5 Conclusions

We investigated the accuracy of gated beam delivery using an Elekta linac with a small number of monitor units delivered in each gating window. Our results suggest that Elekta linacs can deliver gated radiation accurately over a wide range of clinical gating scenarios. A tight gating window (e.g., 17%) should be avoided in free‐breathing gating in order to maintain gating accuracy. These results were confirmed using delivery measurements for SBRT VMAT plans. Additionally, the gated technique could be used for breath hold gating as well. The respiratory gating technique showed better accuracy than the multiple static beam delivery technique as the beam‐hold allows the radiation to reach a stable state more quickly.

## conflict of interest

The authors declare no conflict of interest.
